# Detection of gunshot residues using infrared photography: influence of ammunition type, surface color and blood contamination

**DOI:** 10.1007/s00414-025-03609-9

**Published:** 2025-09-20

**Authors:** Joel Bottoni, Holger Wittig, Thomas Rost, Alexander Schocker, Philipp Wild, Urs Nachbur, Dominique Neuhaus, Lennart Bedarf, Kathrin Gerlach, Eva Scheurer, Claudia Lenz

**Affiliations:** 1 Institute of Forensic Medicine, Department of Biomedical Engineering, University of Basel, Basel, CH-4056 Switzerland; 2 Institute of Forensic Medicine, Health Department Basel-Stadt, Basel, CH-4056 Switzerland; 3 Polizei Basel-Landschaft, Forensic Sciences, Rheinstrasse 25, Liestal, CH-4410 Switzerland; 4 Institute of Forensic Medicine of the University Basel, Pestalozzistrasse 22, Basel, 4056 Switzerland

**Keywords:** Gunshot residues, Infrared photography, Leaded ammunition, Lead-free ammunition, Blood contamination

## Abstract

Detecting gunshot residues (GSR) plays a crucial role in forensic science and forensic medicine by providing important insights into the shooting distance, the shooter, as well as the type of weapon and ammunition used. Detection of GSR on dark surfaces is often impossible on site, and traditional methods such as tape-lift techniques with adhesive films or scanning electron microscope tabs might destroy the GSR pattern at the crime scene during their application. Infrared (IR) photography has proven particularly effective in detecting GSR on dark surfaces, enabling the preservation of the GSR pattern before applying destructive methods. This study aimed to examine how the type of ammunition and the presence of bloodstains affect GSR detection and differentiation using IR photography. 15 types of leaded and 5 types of lead-free 9 mm Luger ammunition were fired onto white cotton fabric and dark-blue denim fabric using the same firearm model, with an additional 14 samples being stained with blood. Resulting GSR patterns varied depending on the ammunition type and fewer GSR particles were visible on denim fabric, even in IR photography, compared to white cotton fabric, but still achieving reliable results comparable to the standard tape-lift method. In addition to the known ability of IR photography to detect GSR from leaded ammunition on clean surfaces, our findings demonstrate for the first time that GSR from lead-free ammunition, as well as GSR on blood-contaminated surfaces can be reliably visualized. In conclusion, IR photography provides a robust, easy-to-use and non-destructive tool for GSR detection, with the distinct benefit of allowing immediate on-site visualization of GSR patterns prior to any laboratory processing. With the use of the custom-made Python script (openly accessible on Github), a quantitative evaluation of GSR comparable to the tape-lift method is a further enhancement in this aspect.

## Introduction

When a firearm is fired, the hammer/striker first hits the primer of the cartridge. This triggers an initial explosion, which leads to a burning of the main charge, the propellant. The pressure from the gases produced during this process propels the bullet through the barrel towards the muzzle, where it exits the weapon [1]. In addition to the bullet, gases from both explosions also exit the barrel through the muzzle due to the expansion of these gases. The resulting cloud of gas contains tiny burned and unburned particles from the primer and the propellant and melted material of the bullet-base, which are referred to as gunshot residue (GSR) particles [2]. These particles settle on nearby surfaces or individuals, such as the shooter’s hands or clothing [3, 4].

Since GSR are often invisible to the naked eye, particularly on dark surfaces or when contaminated with blood, forensic methods for the detection of GSR are of paramount importance in the reconstruction of the crime or the crime scenes. GSR samples are typically collected from locations where their presence is most probable, such as the hands of the suspected shooter, the alleged weapon, the bullet entry site or gunshot wound, and the victim’s clothing. The presence of GSR does not only confirm that a weapon has been fired but might also provide further relevant information. The composition of GSR can reveal the type of firearm and ammunition used at a crime scene, and may also provide clues about the ammunition manufacturers, as they use different primer and propellant formulas [2, 5]. Furthermore, the GSR pattern can be used to determine the type of weapon and the shooting distance, also referred to as muzzle-to-target distance [2, 6, 7]. In addition, testing the hands of a suspect person for GSR can indicate if they have recently fired a weapon [8, 9]. Forensic methods take advantage of the fact that suspicious surfaces can be taped with adhesive films or dabbed with scanning electron microscope (SEM) Tables [10, 11]. Later, lead residues are searched for on the adhesive films and SEM tabs are analyzed under a scanning electron microscope. With that, a reliable detection of the common components of primer formulas and propellants is possible. These include lead, antimony, or barium (or a combination thereof) [2, 12]. Therefore, having a reliable method to make GSR visible before evidence collection - without damaging the pattern - would be highly beneficial.

As modern ammunition is increasingly being produced without lead for environmental and health reasons, new reliable methods are needed to detect GSR, especially in relation to forensic techniques using adhesive films, which can only detect traces of GSR containing lead, antimony or barium [13–17]. The use of alternative light sources, particularly from the invisible light spectrum (e.g. infrared), has already brought numerous advantages to forensic science. IR light can not only render texts from blacked-out or faded documents legible again but has also been demonstrated to reveal dried blood stains and forensic findings on decomposed bodies, such as hemorrhages and tattoos [18–25]. Additionally, IR photography can enhance the visualization of hematomas that are difficult to detect with the naked eye [26–28]. Therefore, it can be concluded that the visibility of GSR could also be enhanced using alternative light sources [29–31]. An early study by M. A. Horvath from 1981 found IR microscopy to be a non-destructive, faster, and more sensitive approach to GSR detection than alternative techniques and hypothesized that GSR may also be detected on bloodstained surfaces [32]. Furthermore, Chaklos et al. demonstrated that bloodstains appear transparent in IR with the use of 800 nm and 900 nm Long-Pass-Filters [33]. Another study conducted in 2012 by Lake et al. showed that by using a stereomicroscope VSC 2000 (Video Spectral Comparator 2000), the contrast between GSR and blood is decreased, in case the fabric is heavily saturated with blood [34]. Unfortunately, the publications did not mention whether the blood was wet or dry when the photographs were taken or when the fabrics were examined under the microscope respectively. Since camera technology and especially complementary metal-oxide-semiconductor (CMOS) chips have continuously improved over the last decade, more sensitive detection has become feasible [35].

In this study, we investigated several novel aspects of IR based GSR detection, exploiting recent advances in camera technology and computational analysis methods. We examined how GSR patterns vary between ammunition types on dark surfaces compared to bright textiles, using the same firearm. Further, we used IR photography to explore the detectability of GSR produced by lead-free ammunition on dark-blue denim fabric in comparison to leaded ammunition. Finally, the effectiveness of IR photography using a 700 nm infrared filter in visualizing GSR on blood-stained surfaces was assessed, evaluating the detectability and visibility of residues despite the presence of either wet or dried blood. Moreover, we developed a custom-made phyton code for an automated, quantitative and standardized evaluation of the GSR (openly accessible on Github), comparable with the tape-lift method.

## Materials and methods

### Experimental setup

**Fig. 1:**
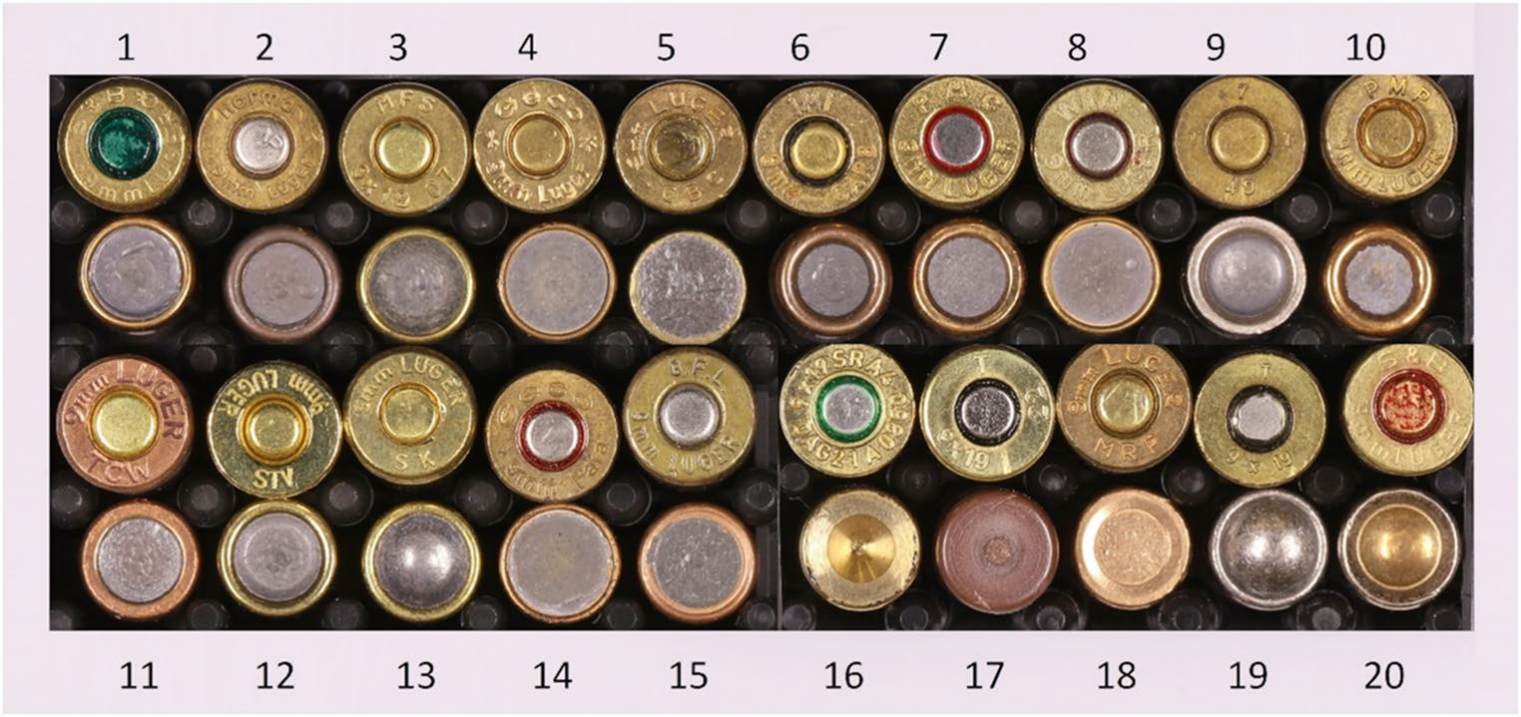
Headstamps of leaded and lead-free ammunition used for all experiments. The number of each ammunition type corresponds to the number in Table 1

**Fig. 2:**
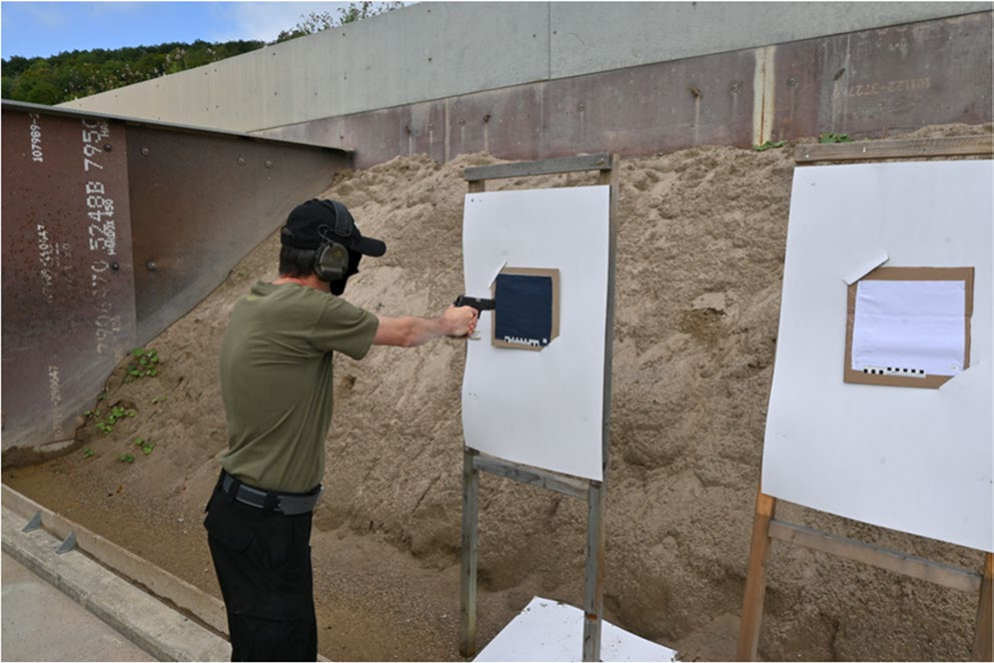
Experimental setup at the shooting range. The picture shows the shooters position before shooting on a piece of dark-blue denim fabric from a distance of approximately 10 cm using a SIG Sauer P220, 9 × 19 mm pistol

15 leaded and 5 lead-free types of ammunition, as described in detail in Table 1, with the corresponding headstamps shown in Fig. 1, were tested. A piece of fabric, either white fabric (100% cotton) or dark-blue denim fabric (100% cotton), was used as trace carrier. Shots were fired directly at the fabric. For each type of ammunition, one piece of each fabric, measuring 25 cm x 25 cm, was attached to a cardboard panel. These cardboard panels were mounted on a stand at the shooting range. Shots were fired at the fabric pieces at an angle of 90° with a distance of approximately 10 cm, representing close-range shots (Fig. 2). Two identical SIG Sauer P220, 9 × 19 mm pistols were used for all shooting experiments, one of which was thoroughly cleaned beforehand and used exclusively with lead-free ammunition.

**Table 1:** Types of leaded and lead-free ammunition used for all experiments

	Manufacturer	Country	Headstamp	
1	Sellier & Bellot	Czech Republic	S&B → Ο 9 mm LUGER	Leaded
2	Norma	Sweden	Norma 9 mm Luger	Leaded
3	Mátravidéki Fémmüvek Sirok	Hungary	MFS 9 × 19 07	Leaded
4	Geco	Germany	Geco * 9 mm Luger *	Leaded
5	Magtech	Brazil	CBC 9 mm LUGER	Leaded
6	Israel Military Industries	Israel	IMI 9 mm CARB	Leaded
7	Precision Made Cartridges	South Korea	PMC 9 mm LUGER	Leaded
8	Winchester	United States of America	WIN 9 mm LUGER	Leaded
9	Thun Munitions Factory	Switzerland	7 T T 49	Leaded
10	Pretoria Metal Pressings	South Africa	PMP 9 mm LUGER	Leaded
11	Tula Cartridge Works	Russia	TCW 9 mm LUGER	Leaded
12	STV Group AS	Czech Republic	STV 9 mm LUGER	Leaded
13	Sellier & Bellot	Czech Republic	SK 9 mm LUGER	Leaded
14	Geco	Germany	Geco 9 mm Para	Leaded
15	Fiocchi	Italy	G.F.L. 9 mm LUGER	Leaded
16	Ruag	Switzerland	DAG21 A 0850 9 × 19 SR A4	Lead-free
17	Ruag	Switzerland	T 22 9 × 19	Lead-free
18	Giulio Fiocchi Spa	Brazil	MRP 9 mm LUGER	Lead-free
19	Ruag	Switzerland	T 9 × 19	Lead-free
20	Sellier & Bellot	Czech Republic	S&B 9 mm LUGER	Lead-free

To prevent a so-called memory effect, in which residual GSR particles from previous shots are expelled from the barrel, a single shot was fired into the sandpit after each ammunition change, before targeting the trace carriers. However, a memory effect from the previous shot with the same type of ammunition was accepted, and even desired, as this ensured that both fabrics experienced a memory effect as equal as possible.

Furthermore, a total of 14 additional trace carriers (7 white fabric and 7 dark-blue denim fabric) were shot with either Geco 9 mm Luger ammunition from Germany (Table 1, Number 4) or PMC 9 mm Luger ammunition from South Korea (Table 1, Number 7). For the first four shots, a sponge soaked with fresh pig’s blood was placed behind the fabric before shooting. For the remaining 10 shots, six pieces of fabric were sprayed with fresh blood before being shot, whereas four pieces of fabric were shot first and then sprayed with fresh blood.

In order to minimize possible cross-contamination, the experiments were carried out on an outdoor shooting range of the police department. Three persons were involved in conducting the experiments. The first person was responsible for loading and unloading the weapon, as well as firing the shot. This person had no direct contact to the trace carriers. The second person attached the new trace carriers to the stands and was responsible for taking the photographs after the shots were fired. The third person was in charge of preserving the trace carriers for subsequent laboratory analysis. Each fabric piece was individually preserved to avoid contamination with GSR from other samples.

### Generation and analysis of photographic data

For each experiment, an identical evaluation process was applied. Immediately after a fabric piece was shot, photographs were taken with two mirrorless cameras (Nikon Z50) equipped with a Nikon Nikkor Z 16–50 mm F 3.5–6.3 VR DX lens.: One camera was used for the visible-light (VL) photographs and the other, modified with a built-in 700 nm infrared filter (modification performed by IRreCams, Dreschvitz, Germany), was used for the IR photographs respectively. Therefore, the original built-in filter from Nikon had to be removed for the IR camera. All fabrics coated in blood were photographed a second time, i.e. one hour after the shot when the blood had dried in the sun at an outdoor temperature of 30° Celsius. The built-in flashlight of both cameras was used for all photographs. Photographs were taken handheld using the following parameters: ISO 125, a shutter speed of 1/125s and varying apertures.

All IR photographs were captured in Nikon’s NEF raw image format (NEF-Format, 12-bit, compressed) and post-processed using Nikon’s NX Studio image editing software (Version 1.7.0) to achieve the highest possible image quality. Each photo was edited following the same steps to ensure comparability. First, the «Picture Control» tab was set to «monochrome». This converted the IR images, which naturally exhibit a red color tone, into greyscale images. This step was necessary to increase the contrast between the dark GSR particles and the background, which appeared bright in IR. The settings «sharpening» and «clarity» were maximized, resulting in a brighter overall image and further enhancement of the microcontrast. The setting «D-Lighting HS» was increased from 0 to 1 to clarify details in well-lit and shadowy areas. The edited raw data were then converted into JPEG files. The VL camera produced photographs in JPEG format that were not further edited for analysis.

Dark-blue denim fabrics that had been shot with lead-based ammunition (15 in total) were further analyzed using tape-lift techniques with adhesive films for comparison. Each piece of fabric was covered with adhesive tape (Netra FILMOLUX^®^ S50) and was subsequently analyzed by a three-step chemical treatment [36]. Frist, the adhesive side of the tape was sprayed with a polyvinyl alcohol layer (PVAL) solution until a thin layer covered the entire adhesive surface. After a short exposure time, the surface was dried with a hairdryer. Second, the same side of the tape was sprayed again with a 2% tartaric acid solution until a thin layer covered the entire adhesive surface. After 60–90 s of exposure time, the surface was dried again carefully with a hairdryer. Finally, the same side of the tape was sprayed with a sodium rhodizonate solution and dried with a hairdryer again. The PVAL solution was prepared by dissolving 100 g of solid polyvinyl alcohol (PVAL) (Sigma-Aldrich) in 500 ml of ultrapure water. The mixture was heated at 90 °C until the PVAL was fully dissolved. The temperature was then reduced to 50–60 °C and 665 ml of denatured ethanol along with 3 g of glycerol (Merck) were added. The tartaric acid solution was prepared by dissolving 25 g of tartaric acid (Sigma-Aldrich) in 1000 ml of ultrapure water, to stabilize the solution and extend its shelf life, 2.5 g of benzoic acid (Sigma-Aldrich) were added. The rhodizonate solution was prepared by dissolving 0.2 g sodium rhodizonate (Sigma-Aldrich) in 50 ml of ultrapure water. After the chemical treatment, the tapes were scanned in order to automatically count individual lead particles.

Both images taken with the standard camera and the processed IR images were first evaluated manually based on their appearance, followed by a quantitative evaluation in which the individual GSR particles were counted. For the manual evaluation, a scale ranging from 0 to 5 points was established using the following criteria: 0 points were assigned if no GSR were visible in the pictures. When GSR were visible, scores ranging from 1 to 5 points were assigned based on the amount of visible particles, using the following increments: minimal, slight, moderate, considerable, and substantial. Each photo was evaluated by three raters, one rater with previous experience in analyzing GSR and two raters without prior experience and without being involved in the practical experiments.

For the quantitative evaluation, a Python program [37] (publicly available) was developed to detect and count GSR particles in each image, ensuring that areas without residues were not mistakenly included. To enhance the visibility of GSR on different types of fabric, Fourier Transform filtering was applied to the images. For white fabric, a high-pass filter was utilized to remove low-frequency components, effectively minimizing the visibility of the underlying fabric pattern and highlighting the GSR particles. In the case of denim, a band-pass filter was employed to eliminate both, low and high-frequency components. This approach was specifically designed to reduce the pattern complexity of the denim fabric, thus, improving the contrast between the fabric and the GSR particles. An intensity threshold was applied to isolate the darkest spots in the image, which correspond to the GSR. This step was crucial for accurately distinguishing GSR particles from the fabric background and to enhance the segmentation process.

The subsequent analysis involved two primary quantifications: the count and the area of GSR particles. The count was determined by identifying all discrete white areas within the segmented image, each representing a GSR particle. For the area calculation, the number of white pixels within these areas was counted. To translate pixel counts into physical dimensions, the actual physical length per pixel was assessed based on the original image calibration. The area of each GSR particle was then calculated using the following formula:

1 $$A\;=\;{(L_{pixel})}^2\;.\;n_{pixel}$$

where A is the area, L_pixel_ the physical length of the pixel and n_pixel_ the number of pixels.

### Analysis of the adhesive tapes

For the quantitative evaluation of the adhesive tapes, the Python program was modified with the following changes: To optimize the contrast between GSR and the background fabric, images were first converted from the RGB (red, green, blue) color model to HSV (hue, saturation, value) colour space. This transformation was particularly beneficial for isolating the value channel, which significantly enhanced the contrast differences between the GSR particles and the fabric particles in the background, facilitating a more effective segmentation.

### Statistical evaluations

To compare the manual assessments of the three raters, the intraclass correlation coefficient (ICC) was calculated for each category (two-way mixed single measures) [38]. This value indicates the degree of agreement among raters in their evaluations. Values between 0.75 and 1 were considered as excellent, values between 0.6 and 0.74 as good, values between 0.4 and 0.69 as fair, and values below 0.4 as poor level of agreement [39]. To evaluate statistically significant differences between particular groups, independent samples t-tests were conducted. A significance level of 0.05 was established to assess statistical significance, meaning that results with a p-value below this threshold would indicate a statistically significant difference between groups [40].

## Results

**Fig. 3:**
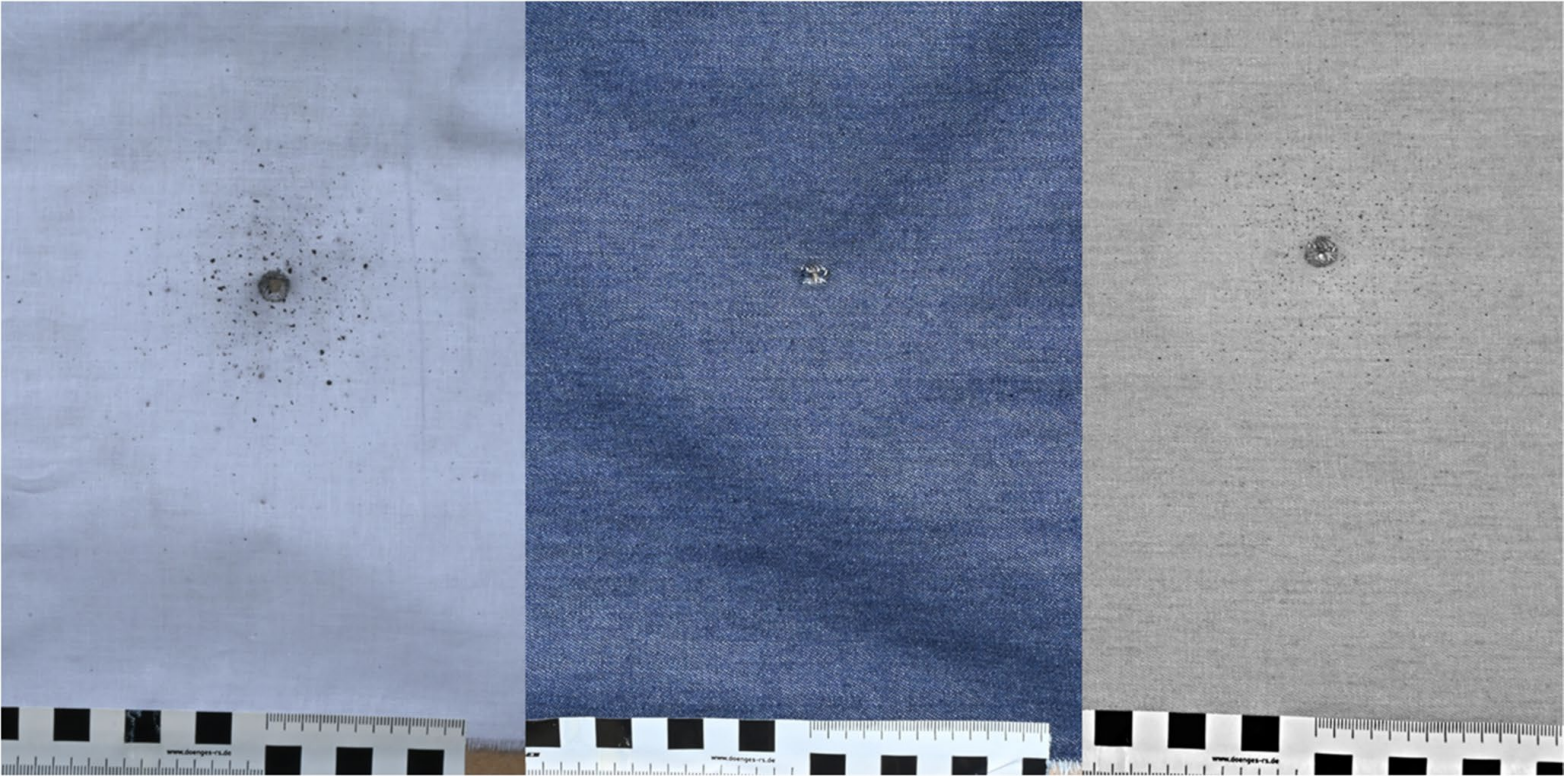
VL photograph of a white fabric (left), VL photograph of a denim fabric (middle) and the corresponding IR photograph of the denim fabric (right). Both fabrics were shot with 9 mm Luger ammunition from Winchester (Table 1/Fig. 1, number 8)

In all VL photographs of the white fabric and all IR photographs of the denim fabric, GSR from leaded ammunition, with rating scores between moderate and substantial for the white fabric and rating scores between minimal and substantial for the denim fabric were visible. Except for one instance, where a rater did not detect GSR in a single IR image of denim fabric, GSR originating from lead-free ammunition were identified in all cases. Figure 3 shows an example of a VL photograph of the white fabric (left) and a VL and IR photograph of a denim fabric (middle and right), both from a shot with Winchester ammunition (Table 1/Fig. 1, number 8).

**Fig. 4:**
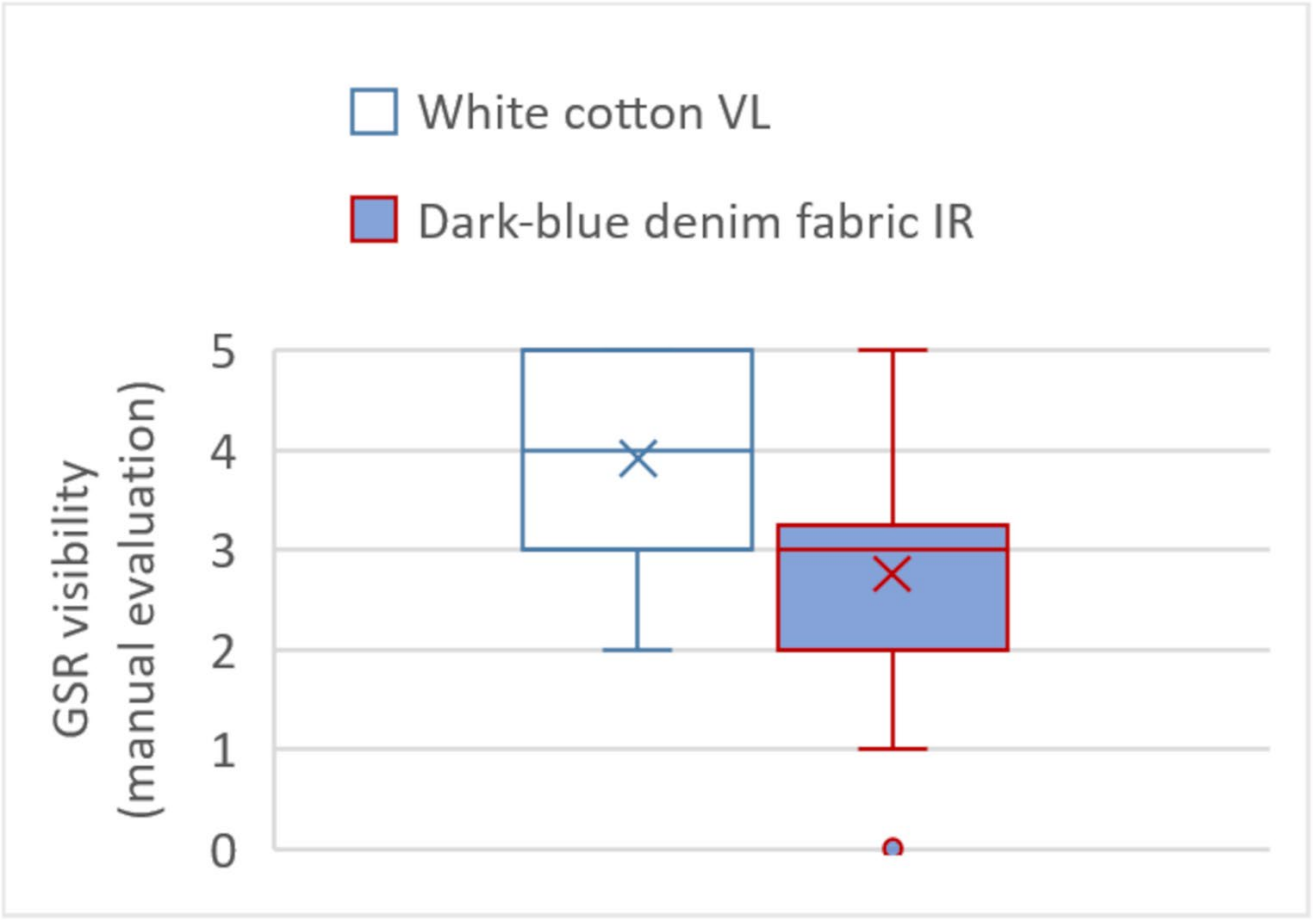
Box-whiskers-plot comparing the visibility of GSR in VL photographs of the white fabric and IR photographs of the denim fabric. Each box contains a total of 60 evaluations, comprising 20 manual evaluations from each of the three raters (0 points = no GSR visible, 1 point = minimal, 2 points = slight, 3 points = moderate, 4 points = considerable, 5 points = substantial)

Figure 4 presents a box-whiskers-plot with the manual assessments of the VL photographs of the white cotton fabric in the left section with a median (marked by an x) of 3.9 points and the manual assessments of the IR photographs of the denim fabric in the right section with a median of 2.8 points. The corresponding t-test comparing the two groups yielded a p-value < 0.001. The calculated ICC of the three raters showed a value of 0.47 (fair level of agreement) for the VL photographs of white cotton fabric and a value of 0.76 (excellent level of agreement) for the IR photographs of the denim fabric. In a single case, no GSR were detected on the denim fabric by one of the three raters.

**Fig. 5:**
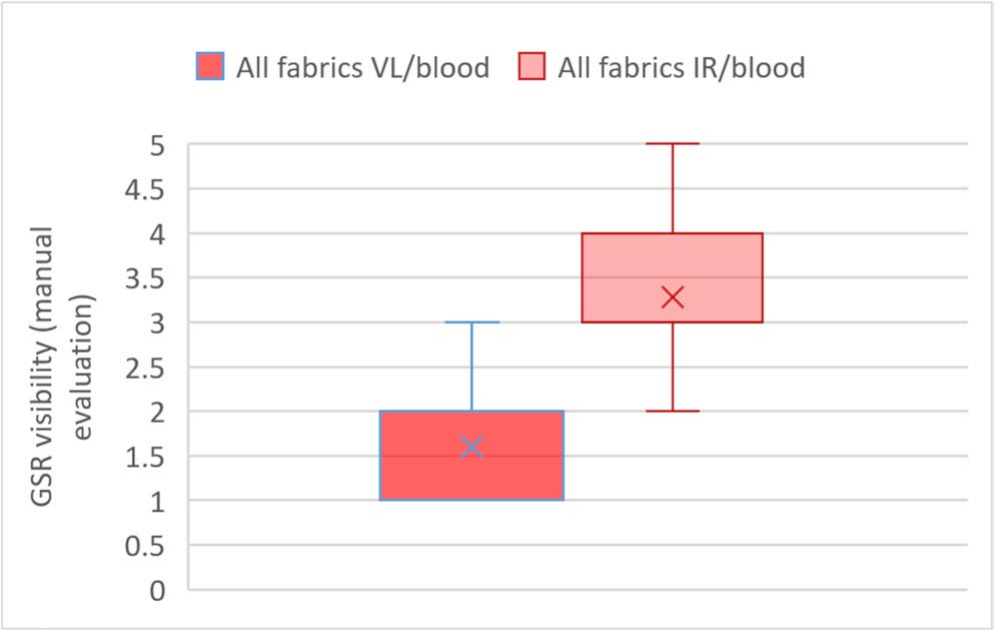
Box-whiskers-plot comparing the visibility of GSR in VL photographs (left) and the corresponding IR photographs (right) of all fabrics coated with blood. Each box contains a total of 42 evaluations, comprising manual 14 evaluations from each of the three raters (0 points = no GSR visible, 1 point = minimal, 2 points = slight, 3 points = moderate, 4 points = considerable, 5 points = substantial)

Figure 5 presents the manual evaluation of the blood coated trace carriers. The evaluation of the VL photographs is shown on the left with a median of 1.6 points, whereas the assessment of the IR photographs is displayed on the right with a median of 3.3 points. The corresponding t-test comparing the two groups yielded a p-value < 0.001 and the calculated ICC showed a value of 0.79 (excellent level of agreement) for the VL photographs and a value of 0.77 (excellent level of agreement) for the IR photographs. No differences were observed in the evaluations between fabrics that were first shot and then bloodstained compared to those that were first bloodstained and then shot.

**Fig. 6:**
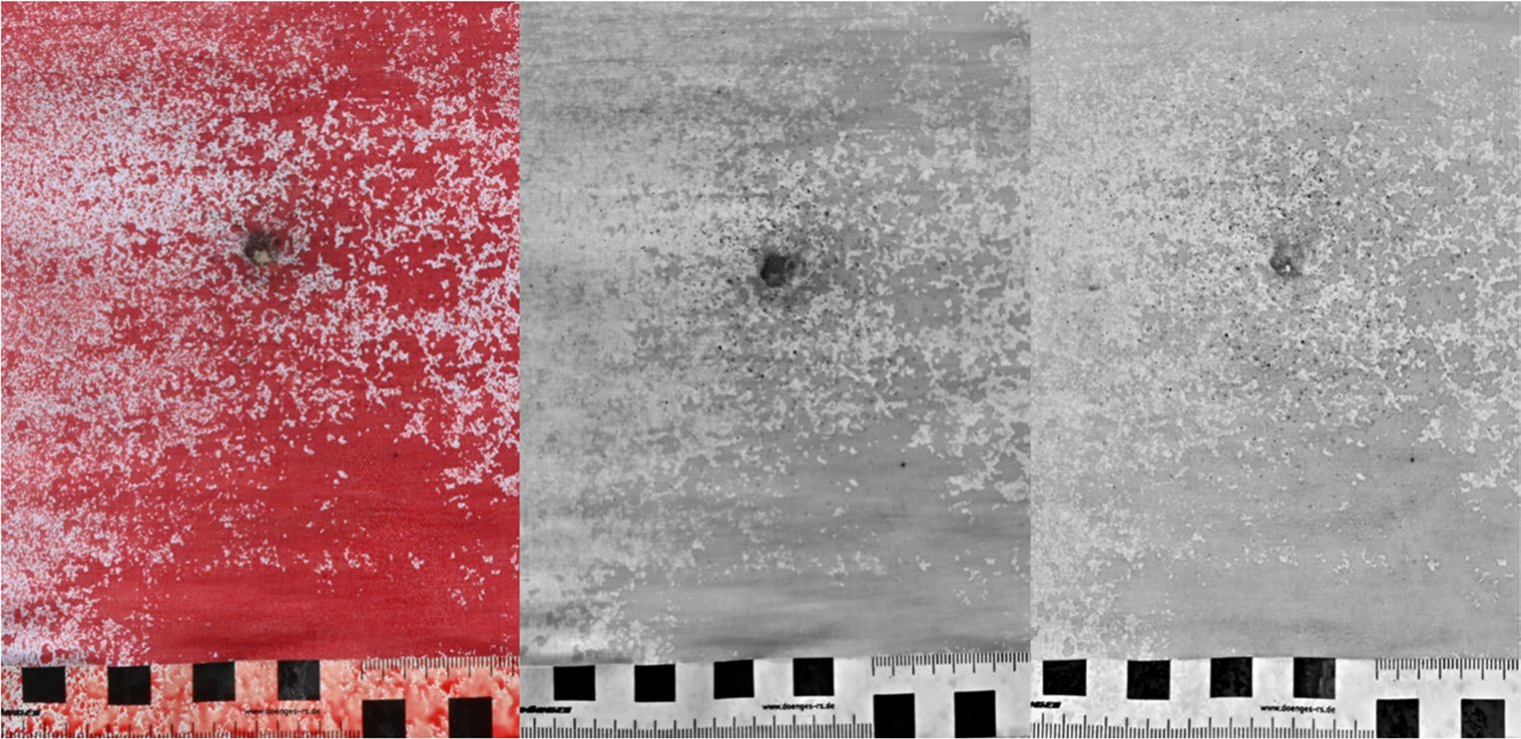
Three photographs of the same white cotton fabric coated with blood after being shot. VL photograph (left), IR photograph taken while the blood was still wet (middle) and IR photograph taken after one hour when the blood had dried (right)

Figure 6 shows three photographs of the same trace carrier - a white cotton fabric sprayed with blood. The VL photograph is presented on the left, while the center and right images represent the IR photographs. In the center image, the blood was still wet at the time of capture, whereas the blood had already dried in the right image. In the VL photograph, a dark cloud of GSR is visible around the gunshot defect, though individual particles cannot be distinguished. In the two IR images, the distinction between bloodstains and GSR is clear. However, the dried blood in the right image appears lighter in IR than the wet blood in the center image, which leads to an increased contrast between blood and GSR.

**Fig. 7:**
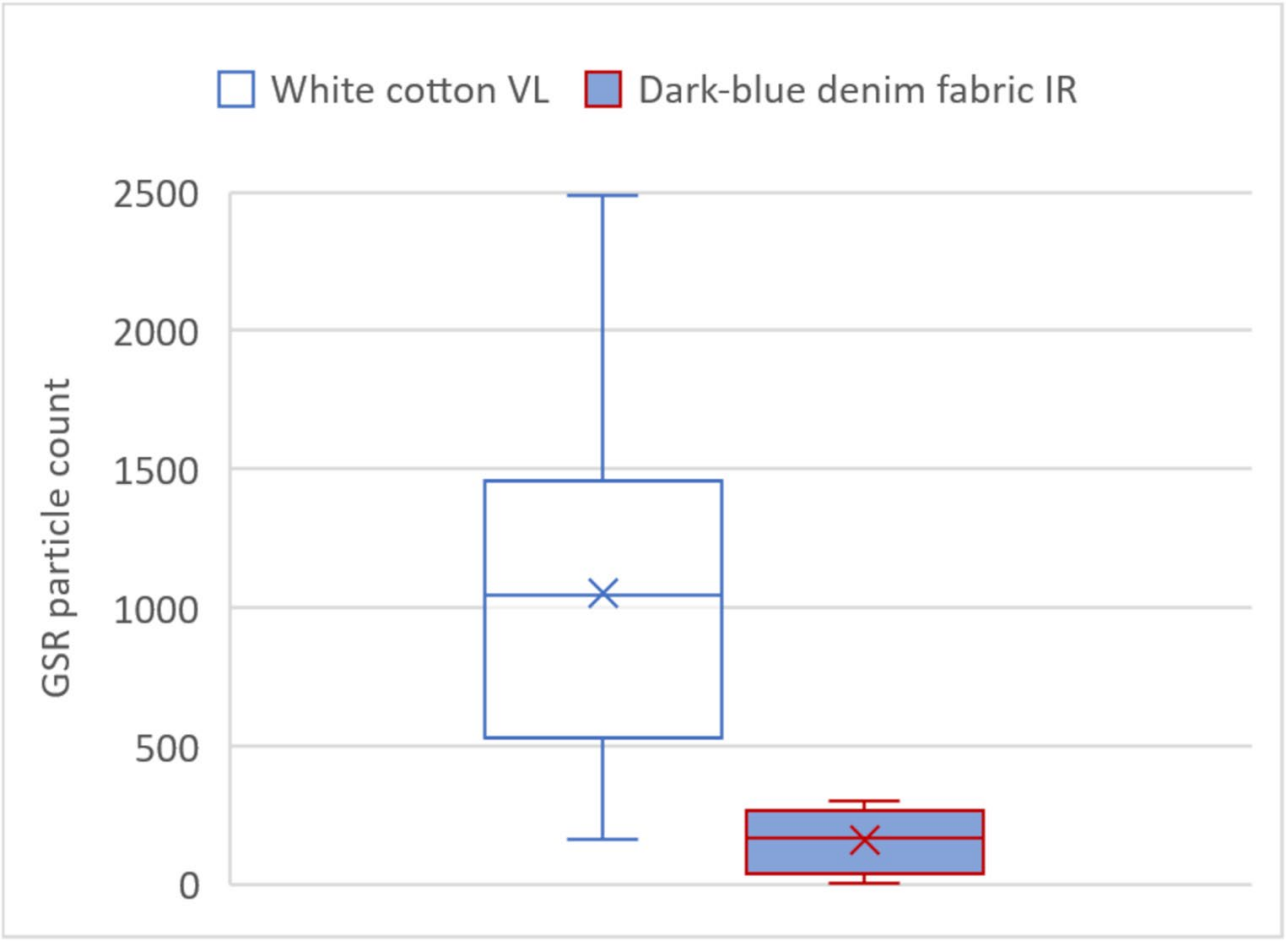
Box-whiskers-plot comparing the count of GSR particles in VL photographs of the white fabric (left) and in IR photographs of the denim fabric (right). Each box contains the count of 20 images (all lead-based and lead-free ammunition types) conducted with the custom-made python script

Figure 7 shows a box-whiskers-plot of the GSR particles counted using the custom Python code. For the white cotton fabric, between 166 and 2468 particles were counted across the 20 VL images examined, with a median of 1053 particles. For the denim fabric, shown on the right, between 7 and 303 particles were counted in the IR images, with a median of 162 particles. The corresponding t-test comparing the two groups yielded a p-value < 0.001.

**Fig. 8:**
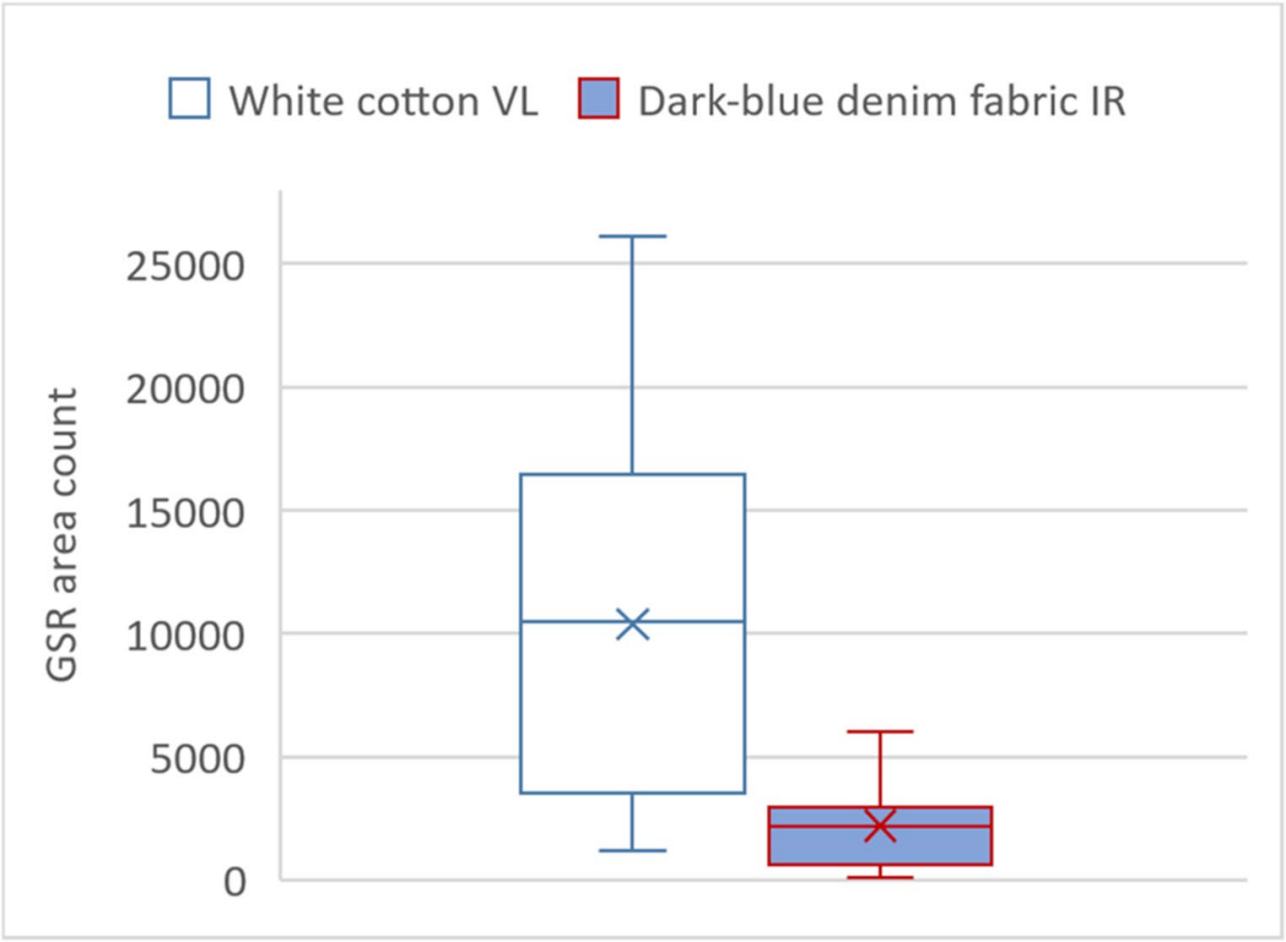
Box-whiskers-plot comparing the area of GSR in VL photographs of the white fabric (left) and in IR photographs of the denim fabric (right). Each box contains the count of 20 images (all leaded and lead-free ammunition types) conducted with the custom-made python script

Figure 8 shows a box-whiskers-plot of the GSR area counted using the custom Python code. For the white cotton fabric, the counted area ranged from 1171 to 26,103 pixels across the 20 VL images examined, with a median of 10,379 pixels. For the denim fabric, shown on the right, the counted area ranged from 76 to 6018 pixels in the IR images, with a median of 2190 pixels. The corresponding t-test comparing the two groups yielded a p-value < 0.001.

**Fig. 9:**
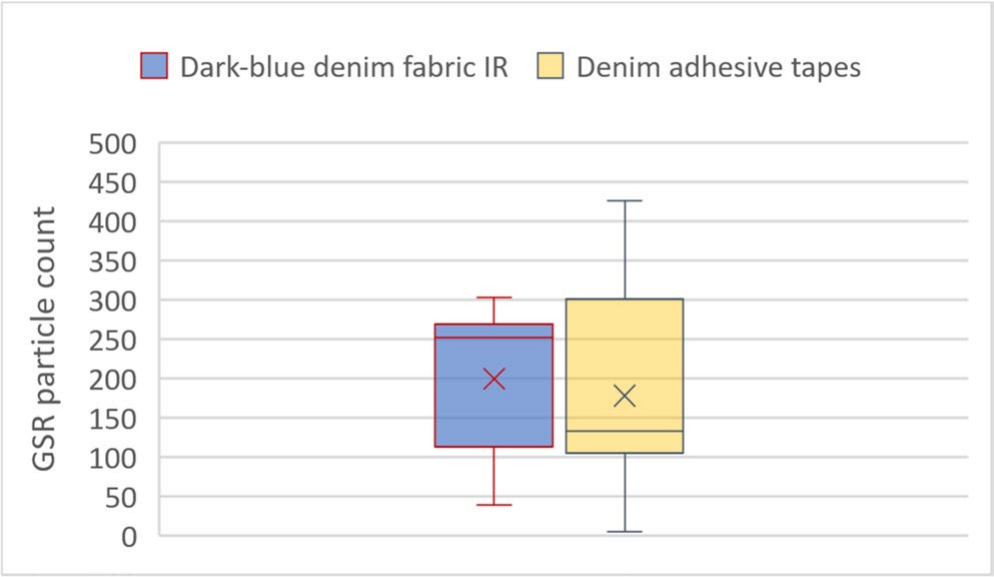
Box-whiskers-plot comparing the automated count of GSR particles in IR- photographs of the denim fabric (left) and the image scans of the corresponding adhesive tapes (right). Each box contains the count of 15 images (all lead-based ammunition types)

As presented in Fig. 9, the automated particle counting revealed no significant difference between the IR photographs of the dark-blue denim fabric shot with leaded ammunition and the chemical developed adhesive tapes of these fabrics. For the 15 adhesive tapes, between 5 and 426 particles were counted with a median of 178 particles. For the IR photographs of the denim fabrics shot with leaded ammunition, between 7 and 303 particles were counted, with a median of 200 particles. The corresponding t-test yielded a non-significant p-value of 0.58.

## Discussion

This study demonstrated that the GSR pattern is dependent on the type of ammunition being fired, even when the same weapon is used. For some types of ammunition, up to 2’468 particles could be detected per shot using the custom Python code, while with other types of ammunition only about 166 particles settled onto the trace carriers. One possible explanation could be the varying compositions of the primers and propellants used by different manufacturers, which have also evolved over time [41]. However, inferring the ammunition type or manufacturer based on the residue pattern alone as can be achieved through the analysis with SEM tabs seems impossible due to the vast diversity of ammunition, resulting in insufficiently distinct residue patterns.

Furthermore, consistent with the findings of Bailey et al. and Barrera et al. [29, 30], it could be demonstrated that GSR can be detected on dark fabric using IR photography. However, with 7 to 303 gunshot particles counted on the denim fabric using the custom Python code, there was a statistically significant difference compared to the GSR detected on the white cotton fabric using VL photography (166 to 2468 particles). Additionally, all three raters independently judged the GSR as more visible in the VL images of the white fabric compared to the IR images of the denim fabric. Even though the denim fabric appears brighter in IR than to the naked eye, a difference in contrast compared to the white cotton fabric is still noticeable. Thus, it can be assumed, that the visibility of GSR varies depending on the brightness of the respective fabric in the IR spectrum. Detecting GSR on dark objects further depends on the ability of the object itself to reflect IR waves. Thus, in case dark fabric completely absorbs IR, it will appear as dark as GSR in IR photography, thereby hindering the detection of GSR [42, 43]. Additionally, the structure of the trace carrier (e.g. smooth versus rough surface) might further influence the deposition of GSR. A study by Ciro et al. suggested that temperature plays a significant role in the deposition of soot particles on textiles, as the temperature gradient between different areas of a fabric can influence how soot particles are attracted to and settle on the surface of the material [44]. GSR deposition may therefore behave in a similar manner. Nevertheless, this study demonstrated that the number of GSR particles visible in the IR images of the denim fabric is in good agreement to the number of particles obtained using the tape-lift method with adhesive films. Therefore, it can be concluded that IR photography achieves equally good results in the detection of GSR as the standard tape-lift method with adhesive films, while also offering the advantage of being a non-destructive and relatively fast technique.

It is well established that the weapon type and the composition of ammunition, particularly the primer, has a significant impact on GSR patterns [5, 8, 45]. However, this study did not include a systematic analysis of the ammunition composition, as its primary objective was not to examine the causes of residue formation, but to evaluate whether IR photography can detect GSR patterns on dark surfaces with special consideration of lead-free ammunition. The reduced IR visibility observed on the dark-blue denim textile compared to the white cotton textile is unlikely to be explained by ammunition composition alone. Additional factors, such as surface texture or wavelength-specific absorption and reflection may contribute to the observed differences [30, 46]. Further research combining controlled analysis of ammunition types with standardized IR imaging protocols is needed to clarify these influences and better understand the determinants of GSR visibility in the IR spectrum.

Although both, GSR and blood absorb IR waves and, thus, appear dark in IR photographs, it could be shown that a distinction between GSR and blood is possible using a 700 nm IR filter. In contrast, the 800- and 900-nm Long-Pass-Filters used by Chaklos et al. rendered bloodstains nearly invisible [33]. The separation of GSR and blood remained clear in our results, although we used a 10 cm standoff, whereas Chaklos et al. fired from 1 inch (2.54 cm) and produced very dense GSR patterns. Compared to the early studies by Horvath and Lake et al., in which the evaluation of photographs was carried out later in the laboratory using microscopy, our approach, based on a digital camera equipped with an integrated 700 nm IR filter, strongly benefits from capturing and analyzing images directly on-site [32, 34]. This makes valuable information immediately available to police and investigators.

In VL photography, it was difficult to identify individual gunshot particles alongside the blood. Additionally, it was observed that in some cases blood droplets reflect the flashlight from the camera, which further limited the distinction between GSR and blood in VL photographs. In IR images, the blood droplets did not reflect the flashlight and did therefore not affect the image quality.

Since the adhesive tape method only reveals lead-containing residues, it cannot detect lead-free GSR. Thus, IR photography provides a distinct advantage, as GSR were clearly visible in both, lead-containing and lead-free samples. However, one limitation of this study is that we investigated lead-free ammunition of only five different manufacturers. Therefore, it would be beneficial to examine a larger variety of lead-free ammunition types from different manufacturers for GSR in IR photography to validate our results. While the detection of GSR using IR provides numerous advantages - being fast, simple, non-destructive and cost-effective - verification through adhesive tapes or SEM tabs remains essential, as it cannot be excluded that other naturally occurring particles might be mistakenly interpreted as GSR in IR photography.

In our quantitative Python analysis, we encountered several challenges specific to the materials tested (white fabric, denim fabric, and adhesive tape) each requiring unique adjustments to the segmentation process due to differences in texture and color characteristics. For instance, the denim fabric required individualized adjustments to the intensity threshold and band filter spectrum in Fourier space to accurately segment the GSR particles. Furthermore, the segmentation process exhibited inconsistencies, such as the omission of light speckles and the unintentional inclusion of darker fabric areas, particularly in GSR regions where material intensities varied. These issues not only highlight the limitations of current segmentation algorithms when dealing with high-contrast materials, but also the risk of distorting GSR distribution, potentially overestimating particle count. To address these inconsistencies, segmentation parameters were fine-tuned to achieve more consistent results across different fabric types (especially between denim and white fabric) and between samples of the same material. This optimization was essential for overcoming the limitations of the segmentation process, ensuring that the results reliably reflect the GSR distribution across various materials. These improvements are crucial for forensic analyses, providing accurate insights into the amount and distribution of GSR on different substrates in crime scene investigations. Therefore, the custom-made python script provides a novel quantitative, objective, automated and standardized method to count GSR particles, with a precision compared to the tape-lift method. The python script represents an effective and non-destructive support in this manner. For this reason, the script is open accessible for future users to include this method in their daily routine or for new study designs [37].

## Conclusion

This study showed that IR photography extends beyond the detection of lead-containing ammunition and can also be applied to the detection of GSR from lead-free ammunition and blood-contaminated surfaces. GSR patterns are influenced by two main factors: the type of ammunition used, with different manufacturers producing varying amounts of GSR and the surface of the trace carrier, which affects the number of deposited GSR particles. We further demonstrated that IR photography, along with the custom-made Python script, effectively detects GSR on dark-blue denim fabric, achieving results comparable to the standard tape-lift method, while being readily available, cost-effective, and non-destructive (corresponding custom-made Python script is open source on GitHub [37]). Notably, IR photography also distinguishes GSR from superimposed blood stains and can detect GSR from lead-free ammunition on dark surfaces. Our findings demonstrate that IR photography offers a suitable tool for the detection of GSR during on-site forensic casework, especially considering the increasing use of lead-free ammunition, which may render the tape-lift method less effective in the future. Further investigations are recommended to study the GSR detection on bloodstained skin, aiming to apply these findings to gunshot wound victims and include a broader range of lead-free ammunition types to validate the findings of this study.

## Data Availability

The datasets generated during and/or analyzed during the current study are available from the corresponding author on reasonable request.

## References

[1] Heard BJ (2008) Handbook of firearms and ballistics: examining and interpreting forensic evidence, 2nd edn. John Wiley & Sons Ltd, Chichester

[2] Vachon CR, Martinez MV (Sep2019) Understanding gunshot residue evidence and its role in forensic science. Am J Forensic Med Pathol 40(3):210–219. 10.1097/PAF.0000000000000483 31233396 10.1097/PAF.0000000000000483

[3] Maitre M, Horder M, Kirkbride KP, Gassner AL, Weyermann C, Roux C, Beavis A (2018) A forensic investigation on the persistence of organic gunshot residues. Forensic Sci Int 292:1–10. 10.1016/j.forsciint.2018.08.036 30265939 10.1016/j.forsciint.2018.08.036

[4] Maitre M, Chadwick S, Kirkbride KP, Gassner AL, Weyermann C, Beavis A, Roux C (2019) An investigation on the secondary transfer of organic gunshot residues. Sci Justice 59(3):248–255. 10.1016/j.scijus.2019.01.007 31054815 10.1016/j.scijus.2019.01.007

[5] Zain ZM, Jaluddin SN, Halim MIA, Subri MSM (2021) The effect of type of firearm and shooting distance on pattern distribution, particle dispersion and amount of gunshot residue. Egypt J Forensic Sci 11(1):10. 10.1186/s41935-021-00225-7

[6] Biedermann A, Taroni F (2006) A probabilistic approach to the joint evaluation of firearm evidence and gunshot residues. Forensic Sci Int 163(1–2):18–33. 10.1016/j.forsciint.2005.11.001 16332419 10.1016/j.forsciint.2005.11.001

[7] Charles S, Geusens N, Nys B (2023) Interpol review of gunshot residue 2019 to 2021. Forensic Sci Int Synerg 6. 10.1016/j.fsisyn.2022.100302 36545124 10.1016/j.fsisyn.2022.100302 PMC9762191

[8] Blakey LS, Sharples GP, Chana K, Birkett JW (Jan2018) Fate and behavior of gunshot residue-a review. J Forensic Sci 63(1):9–19. 10.1111/1556-4029.13555 28543548 10.1111/1556-4029.13555

[9] Minzière VR, Gassner AL, Gallidabino M, Roux C, Weyermann C (2022) The relevance of gunshot residues in forensic science. WIREs Forensic Sci. 10.1002/wfs2.1472

[10] Shrivastava P, Jain V, Suman (2021) Gunshot residue detection technologies—a review. Egypt J Forensic Sci 11. 10.1186/s41935-021-00223-9

[11] Serol M, Ahmad SM, Quintas A, Familia C (2023) Chemical analysis of gunpowder and gunshot residues. Molecules. 10.3390/molecules28145550 37513421 10.3390/molecules28145550 PMC10386329

[12] Frommholz D, Krämer M, Raffelsieper M, Illges H (2011) Kriminaltechnische Methoden zur Aufklärung von Schussdelikten. BIOspektrum 17(2):182–184. 10.1007/s12268-011-0028-0

[13] Moxnes JF, Jensen TL, Smestad E, Unneberg E, Dullum O (2013) Lead free ammunition without toxic propellant gases. Propellants Explos Pyrotech 38(2):255–260. 10.1002/prep.201200021

[14] Weber AK et al (2020) Reduction in lead exposures with lead-free ammunition in an advanced urban assault course. J Occup Environ Hyg 17(11–12):598–610. 10.1080/15459624.2020.1836375 33201787 10.1080/15459624.2020.1836375

[15] Kanstrup N, Thomas VG, Krone O, Gremse C (2016) The transition to non-lead rifle ammunition in Denmark: National obligations and policy considerations. Ambio 45(5):621–8. 10.1007/s13280-016-0780-y 27040101 10.1007/s13280-016-0780-y PMC4980320

[16] McTee M et al (2023) The seasonal threat of lead exposure in bald eagles, Sci Total Environ 889:164256.10.1016/j.scitotenv.2023.164256 37209742 10.1016/j.scitotenv.2023.164256

[17] Fachehoun RC, Lévesque B, Dumas P, St-Louis A, Dubé M, Ayotte P (2015) Lead exposure through consumption of big game meat in Quebec, Canada: risk assessment and perception. Food Addit Contam Part Chem Anal Control Expo Risk Assess 32(9):1501–1511. 10.1080/19440049.2015.1071921 10.1080/19440049.2015.1071921 26161681

[18] Ellen DM, Creer KE (1970) Infrared luminescence in the examination of documents, J Forensic Sci Soc 10(3):159 – 64. 10.1016/s0015-7368(70)70590-2 5510633 10.1016/s0015-7368(70)70590-2

[19] Farrar A, Porter G, Renshaw A (2012) Detection of latent bloodstains beneath painted surfaces using reflected infrared photography. J Forensic Sci 57(5):1190–1198. 10.1111/j.1556-4029.2012.02231.x 22845038 10.1111/j.1556-4029.2012.02231.x

[20] Edelman G, Manti V, van Ruth SM, van Leeuwen T, Aalders M (2012) Identification and age estimation of blood stains on colored backgrounds by near infrared spectroscopy. Forensic Sci Int 220(1–3):239–44. 10.1016/j.forsciint.2012.03.009 22503886 10.1016/j.forsciint.2012.03.009

[21] Barrera V, Bohnert M (2016) Reconstruction of crimes by infrared photography. Int J Legal Med 130. 10.1007/s00414-016-1343-2 10.1007/s00414-016-1343-2 26932868

[22] Finnis J, Lewis J, Davidson A (2013) Comparison of methods for visualizing blood on dark surfaces. Sci Justice 53(2):178–86. 10.1016/j.scijus.2012.09.001 23601726 10.1016/j.scijus.2012.09.001

[23] Clarkson H, Birch W (2013) Tattoos and human identification: investigation into the use of X-ray and infrared radiation in the visualization of tattoos. J Forensic Sci 58(Sep):1264–1272. 10.1111/1556-4029.12237 23879600 10.1111/1556-4029.12237

[24] Starkie A, Birch W, Ferllini R, Thompson TJU (2011) Investigation into the merits of infrared imaging in the investigation of tattoos postmortem. J Forensic Sci 56(6):1569–1573. 10.1111/j.1556-4029.2011.01869.x 21827465 10.1111/j.1556-4029.2011.01869.x

[25] Cullip M, Tran V-C, Ball CG (2021) Tattoo visualization using cross-polarized lighting and infrared photography. Forensic Sci Med Pathol 17(2):350–353. 10.1007/s12024-020-00347-9 33405071 10.1007/s12024-020-00347-9

[26] Bottoni J, Rost T, Wittig H, Bauer M, Scheurer E, Lenz C (2024) Comparison of visible-light and infrared photography for visualizing hematomas postmortem, Forensic Sci Int 366:112300. 10.1016/j.forsciint.2024.112300 39566346 10.1016/j.forsciint.2024.112300

[27] Dubey A, Rupani R, Sharma V, Singh RK, Kumari S, Verma AK (Aug2022) Reflected near-infrared photography: digging deeper into post-mortem examination. J Forensic Leg Med 90:102397. 10.1016/j.jflm.2022.102397 35841695 10.1016/j.jflm.2022.102397

[28] Nijs HGT, De Groot R, Van Velthoven M, Stoel RD (Jan 2019) Is the visibility of standardized inflicted bruises improved by using an alternate (‘forensic’) light source? Forensic Sci Int 294:34–38. 10.1016/j.forsciint.2018.10.029 30447485 10.1016/j.forsciint.2018.10.029

[29] Bailey JA (2007) Digital infrared photography to develop GSR patterns†. Aust J Forensic Sci 39(1):33–40. 10.1080/00450610701324932

[30] Barrera V, Fliss B, Panzer S, Bolliger SA (2019) Gunshot residue on dark materials: a comparison between infrared photography and the use of an alternative light source. Int J Legal Med 133(4):1115–1120. 10.1007/s00414-018-1965-7 30430255 10.1007/s00414-018-1965-7

[31] Kersh KL, Childers JM, Justice D, Karim G (2014) Detection of gunshot residue on dark-colored clothing prior to chemical analysis, J Forensic Sci 59(3):754 – 62. 10.1111/1556-4029.12409 24634985 10.1111/1556-4029.12409

[32] Horvath MA (1981) Interpretation of gunshot residue patterns using infrared microscopy. AFTE J 13(1):21–31

[33] Chaklos DL, Davis AL (2005) Visualization of gunpowder residue patterns using a digital infrared camera and optical filters. AFTE J 37(2):117–122

[34] Lake H, Milam A, Waskel J (2012) Visualization of gunshot residue patterns using a video spectral comparator 2000. AFTE J Vol 44(1):29–37

[35] Advancements in CMOS Sensor Technology (2025) https://www.edmundoptics.com/knowledge-center/trending-in-optics/advancements-in-cmos-sensor-technology/. Accessed 30 Jul 2025

[36] Werner D, Gassner A-L, Marti J, Christen S, Wyss P, Weyermann C (2020) Comparison of three collection methods for the sodium rhodizonate detection of gunshot residues on hands. Sci Justice 60(1):63–71. 10.1016/j.scijus.2019.09.004 31924290 10.1016/j.scijus.2019.09.004

[37] Domineuq (2025) SchmauchQuant. https://github.com/Domineuq/SchmauchQuant. Accessed 20 Apr 2025

[38] McGraw K, Wong S (1996) Forming inferences about some intraclass correlation coefficients. Psychol Methods 1(1):30–46. 10.1037/1082-989X.1.1.30

[39] Cicchetti D (1994) Guidelines, criteria, and rules of thumb for evaluating normed and standardized assessment instruments in psychology. Psychol Assess 6:284–290. 10.1037/1040-3590.6.4.284

[40] Mishra P, Singh U, Pandey CM, Mishra P, Pandey G (2019) Application of student’s t-test, analysis of variance, and covariance. Ann Card Anaesth 22(4):407–411. 10.4103/aca.ACA_94_19 31621677 10.4103/aca.ACA_94_19 PMC6813708

[41] Chang KH, Jayaprakash PT, Yew CH, Abdullah AFL (2013) Gunshot residue analysis and its evidential values: a review. Aust J Forensic Sci 45(1):3–23. 10.1080/00450618.2012.691546

[42] Pearl MR, Brooke H, McCutcheon JN, Morgan SL, Myrick ML (2011) Coating Effects on Mid-Infrared Reflection Spectra of Fabrics. Appl. Spectrosc. 65(8):876–884. 10.1366/10-06137 21819777 10.1366/10-06137

[43] Brody H, Quynn RG Measurement of opacity in fibers. Text Res J, 35, 6, pp. 524–529, 1965/06/01 1965, 10.1177/004051756503500606

[44] Ciro WD, Eddings EG, Sarofim AF (2006) Experimental and numerical investigation of transient soot buildup on a cylindircal container immersed in a jet fuel pool fire. Combust Sci Technol 178(12):2199–2218. 10.1080/00102200600626108

[45] Schwoeble AJ (2000) Current methods in forensic gunshot residue analysis. C. P. LLC, Florida, US, pp 11–12

[46] Jian Z, Dayu B, Miaomiao Z (2020) Research on detection of gunshot residues on textile by infrared spectroscopic imaging, IOP conference series: materials science and engineering, vol. 738. 10.1088/1757-899X/738/1/012002

